# Is it Ethnic Fractionalization or Social Exclusion, Which Affects Social Cohesion?

**DOI:** 10.1007/s11205-015-1205-1

**Published:** 2015-12-19

**Authors:** Irene van Staveren, Zahid Pervaiz

**Affiliations:** 1 Economics of Development and Emerging Markets, Institute of Social Studies (ISS), Erasmus University Rotterdam, PO Box 29776, 2502 LT The Hague, The Netherlands; 2 Department of Economics, National College of Business Administration and Economics (NCBA&E), Lahore, Pakistan

**Keywords:** Social cohesion, Ethnic fractionalization, Economic growth, Cross-country analysis, Social exclusion, Minorities, Developing countries, O11, Z13

## Abstract

The theory about missing links of economic growth often lags behind the empirical estimations of such links. A consensus has emerged that ethnic fractionalization has a negative impact on growth, also when controlled for income inequality. Often, although implicitly, the assumed channel is social cohesion. We analyse the effect of fractionalization on social cohesion with a different inequality measure, namely a social measure of inequality: the Inclusion of Minorities Index. Our results indicate that it is *social exclusion*, which reduces social cohesion, rather than *diversity as such*. We conclude that future studies of social cohesion and its relation to growth may benefit from using measures of social exclusion next to ethnic diversity.

## Introduction

For 20 years, the missing link of economic development in general and economic growth in particular has been sought in intangible factors, such as religion, social capital, governance, and institutions (see, for example, World Bank 1998; Acemoglu and Robinson 2012). This has resulted in the construction of many interesting proxy-variables, and even more growth regressions. But the theorization of the missing links between the new variables and growth success lags behind the impressive flow of empirical estimations. Whereas the edifice of estimation methods becomes bigger, the theoretical foundations remain relatively weak.

Most theoretical progress has been made around two missing links: social capital and institutions (Acemoglu and Robinson 2012; Dasgupta and Serageldin 1999; Grootaert and van Bastelaer 2002; Nooteboom 2002; Putnam 2000; Rodrik 2003; Woolcock 1998; World Bank 1998). Both concepts have triggered rich academic debates about the *what* and *how* of these links for development outcomes, including critiques and scepticism about the policy relevance, in particular about social capital and historically rooted institutions (Baron et al. 2000; Dolfsma and Dannreuther 2003; Field 2003, Fine 2001; Harriss 2001).

Interestingly, the literature on the two missing links is currently converging. Next to trust in people, social capital research includes trust in institutions (Cook 2009; Hooghe 2003; Scrivens and Smith 2013; Williamson 2009). And researchers of institutions recognize the importance of informal institutions, the non-codified norms, beliefs, and attitudes, next to formal institutions (Hillenkamp et al. 2013; Morrisson and Jütting 2005; Williamson 2009). This convergence has led, among others, to attention to another intangible variable, namely social cohesion. Social cohesion is sometimes also characterized as bridging social capital (between groups and individuals with different social identities) as compared to bonding social capital (within groups but not with outsiders). We will not go into the distinctions between the economic concept of bridging social capital and the sociological concept of social cohesion here. What matters is that the literature on the missing links of economic development has begun to recognize the crucial role of the social-level, or meso-level, variable of social cohesion. This factor is considered to provide stability and trust and cohesion to societies, with economic benefits in terms of low transaction costs, collective action, contributions to public goods, and conflict mediation (Alesina and La Ferrara 2005; van Staveren and Knorringa 2008; Christoforou and Davis 2014).

A much less theorized missing link in development economics is diversity, often measured as ethnic fractionalization or ethnic polarization. The link to economic growth has been theorized rather loosely, however. The link is generally understood in two ways. First and most importantly, ethnic fractionalization is considered as a cost of overcoming differences for exchange and the supply of public goods, and second as a benefit of variation leading to productivity increases and trade (see, for example, Alesina and La Ferrara 2005; Putnam 2007). Putnam argues that, despite negative effects of ethnic diversity in the short run, diversity will have benefits in the long run—if we learn how to handle diversity. Alesina and La Ferrara argue instead that there is a trade-off between the costs and benefits of diversity and that the costs are generally more pronounced than the benefits. For example, recent experimental results on the effect of groups in social behaviour indicates that the presence of social groups reinforces ‘us-them’ behaviour, with negative overall outcomes (Hargreaves Heap and Zizzo 2009).

The empirical results of growth regressions including ethnic fractionalization almost always show a statistically significant negative effect on growth (see, for example, Easterly and Levine 1997; Alesina et al. 2003). We will go deeper into this debate in the literature review below. There is clearly some empirical support for the hypothesis that the costs of diversity are stronger, or at least more salient in the short and medium term, than the benefits. However, critics have argued that it is not diversity itself but the inequality that often goes along with it which drives this result (see, for example, Baron et al. 2000; Fine 2001; Harriss 2001; Dolfsma and Dannreuther 2003; Casey and Owen 2014). Hence, they argue that the analysis should also address inequality between groups. But the empirical results generally remain negative when controlled for income inequality: ethnic fractionalization and polarization keep their statistically significant negative effects on growth even when the estimations control for income inequality (often measured with the Gini coefficient). This persistent empirical result has given rise to a consensus about ethnic diversity as having a predominantly negative effect on growth, even when controlled for inequality. Moreover, the empirical effect is generally found to be *direct*, rather than through investment or trade or other channels—presumably affecting growth through the cracks caused in social cohesion, leading to suspicion and fear, corruption, riots, conflict or violence between social groups (see, for example, Kanbur et al. 2011, and their symposium on ethnic diversity and ethnic strife published in *World Development* vol. 39 issue 2, 2011).

Theoretically, however, the result is difficult to explain. First, it would imply that the costs of diversity always override the benefits of diversity. Second, it would imply that the result is not driven by inequalities between groups, but by the very diversity itself. This is puzzling. Diversity is generally regarded as positive for markets, as opposed to monopolies, over-regulation, and lack of innovation. Moreover, the law of requisite variety, suggesting that more complex decisions require more variation in decision-making teams, has been supported empirically in research about company boards and team diversity (van Emmerik et al. 2008; Bear and Woolley 2011; Desvaux et al. 2007; Zenger and Folkman 2012). Hence, there is no theoretical ground why diversity would be inherently bad for economic growth. The Netherlands, for example, was a country of wide religious diversity in the Golden Age, attracting skilled migrants and providing refuge to intellectuals like Pierre Bayle, a French Huguenot and Baruch de Spinoza, a Portuguese Jew. Religious diversity went together with tolerance and thereby helped investment, trade and innovation, and in particular trust and cooperation, the driving forces behind the Dutch Golden Age. At the same time, conflict, distrust, alienation, and discouragement through discrimination are likely to be bad for economic growth. They hamper productivity, innovation, collective action and trust. The examples abound, from Pakistan to Rwanda, and from Guatemala to South Africa. But is this due to an increasing number of ethnic groups? Is ethnic fractionalization, a measure for the number of ethnic groups, a good proxy for conflict and distrust in a society? And would diversity in a society automatically lead to alienation of groups and discouragement for certain groups to invest in their human capital or to be entrepreneurial?

We argue that diversity as such, with its potential economic benefits, must be distinguished from the social exclusion that may very well go together with an increase in groups of different ethnic, religious, or linguistic backgrounds. But the way to test this is not through a measure of income inequality as a control variable, because income inequality is a vertical inequality, whereas social groups are an expression of horizontal inequality (Stewart 2008). Instead, the type of inequality between social groups is horizontal and can be characterized as social exclusion. We therefore argue that what matters for development is not so much the number of groups and vertical inequalities, but how groups relate to each other. Do they tolerate and respect each other or do they discriminate each other and fight over scarce resources? Is there generalized trust of others or only in one’s own group? Are group members willing to interact with others? Do they feel listened to and accepted as members of society? In other words: do groups contribute to social cohesion or do dominant groups disrupt social cohesion through the social exclusion of other social groups?

In this contribution, we argue that diversity may perhaps help explain variation in economic growth between countries. But if so, it is likely due to social exclusion of social groups, with a negative effect on growth. Social exclusion is likely to have a direct negative effect on growth by eroding social cohesion. So, growth equations with ethnic diversity among the explanatory variables should include the horizontal inequality of social exclusion, rather than the vertical inequality of income or wealth distribution, we suggest. But in this article, we explore the relationship between social cohesion on the one hand, and ethnic diversity and social exclusion on the other hand and we do not run growth regressions. We focus on the *mechanism* linking ethnic diversity to growth, namely social cohesion. Our estimation results indicate that ethnic diversity has no negative effect on social cohesion when it is controlled for social exclusion.

The rest of the article is structured as follows. The next section provides a literature overview and the model. In the section thereafter, we discuss the methodology of our estimations, and we introduce the variables of social cohesion and social exclusion and the data. The following section shows the results. The section after that discusses the results in relation to the model and the literature, and we end with a conclusion.

## Literature Review and Model

The empirical literature on the development effects of ethnic diversity focuses on growth. It recognizes indirect effects, through key variables such as trade, investment and public expenditures, and direct effects, which are referred to in terms of social cohesion and its characteristics such as generalized trust and collective action.

The prelude to the debate was Perotti’s analysis (1996), which demonstrated that socio-political instability has a negative effect on growth, through distributional mechanisms, but that ethnic fractionalization has no statistically significant effect on socio-political stability. In a joint paper by Alesina and Perotti (1996), the indirect channel through investments was analysed. The authors concluded that income inequality has a negative effect on investment, and that ethnic fractionalization has no effect on investment. Hence, they did not find an indirect effect of fractionalization. The turning point in the debate came with an influential article by Easterly and Levine (1997), focusing on Africa. They analysed long-run growth effects of ethnic diversity and found a large and statistically significant negative effect of ethnic fractionalization on Africa’s economic growth between 1960 and 1990. They found both a direct effect and an indirect effect through public choices, which they attribute to polarization around public goods, financial repression, and an overvalued exchange rate favouring elite groups. They conclude that their results “lend support to theories that interest group polarization leads to rent-seeking behaviour and reduced the consensus for public goods, creating long-run growth tragedies.” (Easterly and Levine 1997, 1241). However, they confused diversity (their measure) with polarization (the theoretical concept they use), and they assumed that a larger variation of ethnic groups in a society is a proxy measure for conflicts of interest in a society.

A next key contribution to the debate came from a historical analysis of migration to the New World, by Sokoloff and Engerman (2000). Comparing paths of development in the America’s, the authors found that in some countries, elite groups managed to shape institutions to their own advantage, at cost of the rest of the population and with a negative development effect. Countries with more homogeneity at the beginning of their development, suffered less from such elite capture than countries with more diversity, they noted. What they did not recognize is that it is not so much diversity itself but powerful elites, excluding others from certain benefits, which matter. This implication was picked-up by Keefer and Knack (2002), who analysed the effect of polarization on growth. They measured polarization as the extent to which a country has one or more dominant groups. They found that polarization has a statistically significant negative effect on growth, through insecure property rights, and that it considerably reduced the negative effects of income and land inequality on growth.

Alesina et al. (2003) take the debate further with the development of a new dataset on ethnic, linguistic, and religious fractionalization, as well as the use of a polarization measure. They replicate the study by Easterly and Levine (1997) with the new data and a larger group of countries and confirm the result that both ethnic and linguistic fractionalization has a negative effect on growth, but religious fractionalization not. And they find that their fractionalization measures perform a bit better than the polarization measure they introduced. But they caution that their variables correlate highly with other potential explanatory variables, so that they conclude “in the end, one has to use theory and priors to interpret our partial correlations” (Alesina et al. 2003, 183). In other words, they indicate that the literature has made progress in terms of variables and data, but still lack sufficient theorization. Alesina and La Ferrara (2005) follow-up with a contribution that pays more attention to theory, as was referred to already in the introduction. “The potential benefits of heterogeneity come from variety in production. The costs come from the inability to agree on common public goods and public policies” (Alesina and La Ferrara 2005, 769). They are the first development economists comparing the empirical literature on development with that on ethnic diversity at the local level in developed countries. In their study of American communities, they find a negative effect of ethnic diversity only in poor communities, and they find that linguistic and lifestyle diversity are associated with better outcomes. In an extensive last section titled “open questions”, they ask important questions, in particular about measurement. Here they recognize that it is difficult to determine which diversity dimensions are politically or economically salient, and whether it is diversity as such or polarization, which should be measured.

The empirical debate on the last point mentioned by Alesina and La Ferrara (2005) is taken further by Montalvo and Reynal-Querol (2005) who compare the direct and indirect effects of fractionalization and polarization. They conclude that the indirect effects of fractionalization on growth are limited, but the direct effects strong, just as strong as the indirect effects of polarization through investment, government expenditures and conflict. These results are confirmed by Gören (2014). Papyrakis and Mo (2014) find that both ethnic fractionalization and ethnic polarization affect growth through the corruption channel. Desmet et al. (2012) find that ethno-linguistic fractionalization has a negative effect on public goods and growth, while polarization is largely unrelated to growth.

As we have indicated in the introduction, an important part of the debate concerns the role of inequality. This issue has recently been taken-up by Casey and Owen (2014) in a sophisticated empirical analysis. They use income and wealth inequality next to fractionalization as endogenous explanatory variables in their estimations of growth, institutions, and schooling. They find that fractionalization has a negative effect while income inequality has a positive effect. But they recognize that the analysis should include other forms of inequality, emphasizing elite capture at the cost of other groups. “Our results suggest that it may be more appropriate (…) to explicitly include elites that are identified by their membership in specific ethnic groups and not solely based on an income class” (Casey and Owen 2014, 42). This is precisely what our analysis elaborates, going beyond income inequality to social group inequality.

The review of the growth literature on ethnic diversity has two important implications. First, the influence of ethnic diversity on growth seems to be *direct* rather than *indirect*. This implies that the channel from ethnic diversity to growth is not through economic variables but through the social-economic *process* of interaction between economic agents—through factors such as trust, cooperation, corruption, prejudice and social conflict. These factors are generally captured by the concept of social cohesion, referring to the extent to which communities are harmonious and connected. Second, it is important to control for inequality, but income inequality is probably not the best inequality measure.

These two points are also the major points raised in two critical contributions to the literature. In the first one, Fedderke et al. (2014) the point is made with a historical case study of South Africa, that over time ethnic, linguistic, and religious diversity reflect less essentialist group differences and more differences out of a choice. The authors conclude that the trends in the data are “due not so much to changes in underlying cleavages in South African society, but to changes in the nature of identity formation. In particular, the evidence is consistent with a shift of identity formation that is based on ‘essentialist’ linguistic (ethnic) roots, to one which is choice-based” (Fedderke et al. 2014, 275). Another critique of the consensus emerging in the empirical literature is from Andrey Shcherbak (2012) in a working paper, reporting of an exploratory analysis, which finds that it is not diversity as such, which effects upon development outcomes. But rather, it is the extent of *tolerance* of diversity, which shows a *positive* effect on innovation and investment. This suggests, in line with what Putnam (2007) already suggested as discussed above, that it is not diversity, which matters, but the extent to which a society deals with diversity, which is key for economic growth.

Two studies at the local level of developing countries support this perspective. One of these studies is on Kerala, the Indian state with relatively high social-economic development despite high ethnic fractionalization. The study “shows how the cohesiveness of a political community, a subjective feeling of belonging to a common polity, which need not be related to ethnic demography, can be a driver of public goods provision and levels of social development” (Singh 2010, p. 290). Furthermore, the study concludes “how a sense of one-ness among ethnic groups fosters support for collective welfare and makes residents more likely to work together to monitor social services” (idem). The other local level study is on 59 villages across Gambia. The authors “find little conclusive evidence that ethnic diversity plays a role on shaping the structure of economic networks or that people from ethnic minorities are less likely to engage in economic exchanges” (Arcand and Jaimovich 2014, p. 20).

The implications from the empirical development literature also resonate with a similar debate at the local level of communities in developed countries. We briefly summarize the major insights of this parallel literature, to which Alesina and La Ferrara (2005) already connected, as we noted above. We do this because these are very detailed studies, focusing on the relationships between ethnic diversity, social exclusion and social cohesion—precisely the variable of interest for our cross-country analysis. A study on British neighbourhoods by Letki (2008) compares the effect of ethnic diversity with neighbourhood social status. She finds that the eroding effect of diversity on social cohesion is limited when controlled for neighbourhood status. Her study points at the role not of income inequality, but of social deprivation at the neighbourhood level—a horizontal inequality. The results by Letki are confirmed by Meredith Greif (2009) for Los Angeles, by Tolsma et al. (2009) for the Netherlands, and by Silver and Messeri (2014) for New York City. They all point out that it is not diversity as such but socioeconomic deprivation of particular social groups, which results in negative effects on community social cohesion.

Another study with community data from the US and Canada by Stolle et al. (2008) has disentangled the effect of diversity on community social cohesion from the way different types of people connect to each other. The authors found that white majorities in ethnically diverse neighbourhoods experience less negative effects on trust in their neighbourhood when they regularly talk with their neighbours. So, apparently, negative effects of ethnic diversity are reinforced by stereotypes between groups and can be mediated by social ties. “It is diversity without contact that is most problematic”, the authors state (Stolle et al. 2008, 61). This points in the direction of a role for horizontal inequalities between ethnic groups, rather than vertical inequalities. The results for North America are confirmed in a working paper by Algan et al. (2013) about neighbourhoods in France.

**Fig. 1:**
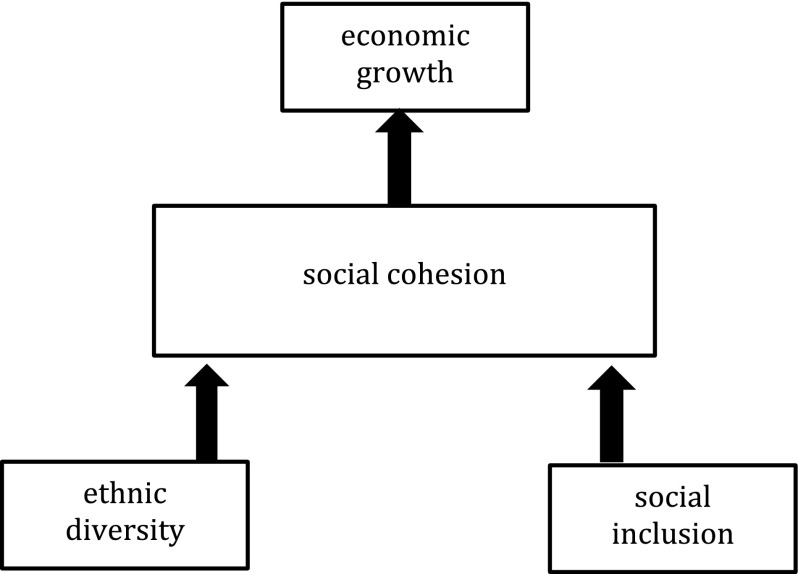
Social economic model of ethnic diversity and economic growth

The community-level empirical research points out that the effects of ethnic diversity on social cohesion in communities is largely due to socioeconomic deprivation and the lack of interaction which is necessary to overcome negative stereotypes and distrust. Together with the two key findings from the most recent empirical development literature on ethnic diversity (a direct effect rather than indirect through economic variables, and the role of social inequalities rather than income inequality), we suggest that the often observed negative effect of ethnic diversity on social cohesion in developing countries is probably not due to diversity as such. It is not so much the number or size of ethnic groups which matter, but rather the way ethnic groups are socially positioned and its individuals relate to each other, which affect social cohesion, and thereby economic growth processes and growth rates. Figure 1 pulls these insights together in a social economic model of ethnic diversity and economic growth.

Social economics regards individuals as embedded in social groups (Davis 2003). This makes their agency influenced by their group membership, but not determined by it, because of individual reflection on the group’s identity and social norms. At the same time, groups are not treated the same by other agents, other social groups, firms or the state. The interaction between members of different groups occurs for example through stigmatization and statistical discrimination, which treat all members of a group in the same way, irrespective of their individual characteristics. So, by being a member of a social group, agents may experience social exclusion, simply because of their group membership and the attributes that non-members ascribe (rightly or wrongly) to this group. Hence, the social economic model of ethnic diversity and economic growth distinguishes social groups (with their own and ascribed social identities) and social exclusion (constraining institutions and individual behaviour against members of particular social groups).

The social economic model puts social cohesion in the middle as the mechanism through which the direct effects of ethnic diversity and social inclusion are mediated in economic growth processes. The economic effects of social cohesion are assumed to be positive, in line with not only the literature discussed above but also based on the literature on social capital and social cohesion as referred to in the introduction: more socially cohesive societies are likely to have higher growth rates. The effects of ethnic diversity and social inclusion are also assumed to be positive. Diversity is expected to have a positive effect on social cohesion, because when controlled for the social exclusionary effects of groups, the potentials of diversity for economic growth remain. And since we expect a negative effect of social exclusion, we assume that social inclusion has a positive effect on social cohesion.

The hypothesis emerging from the social economic model of ethnic diversity and economic growth that we will test below is that both ethnic diversity and social inclusion have a positive effect on social cohesion. We will only test the bottom part of the model in this analysis, focusing on the direct effects of diversity and exclusion on social cohesion as the mechanism linking diversity to growth.

## Methodology and Data

We use data for all developing countries, as much as they are available. See Table 6 in the Appendix for a country list. We use the same data as Alesina et al. (2003), and many other studies, for ethnic diversity. So, our key explanatory variable is Ethnic Fractionalization (EF), ranging from 0 to 1, with 0 referring to complete ethnic homogeneity of a country’s population, and 1 to extreme ethnic diversity. For the robustness test we also use, from the same data source, Linguistic Fractionalization (LF) and Religious Fractionalization (RF), also running between 0 and 1. All three measures are clear measures of diversity: the more groups, the higher the score.

Our other key explanatory variable is the index for Inclusion of Minorities (IM), also between 0 and 1, with 1 referring to full inclusion of all groups in society, and hence, no horizontal inequalities between social groups. The data for Inclusion of Minorities are available in the online database Indices of Social Development, from the Institute of Social Studies. The database Indices of Social Development is an online freely available database with six indices that are based on existing data from publicly available indicators. Each index is constructed through a ranking exercise, using the matching percentiles method, and is available from the website with standard errors provided (for an introduction of the database, see Foa et al. 2013). For details and methodology of the database, as well as background papers, the data, and working papers, please visit the database website.^1^ Table 1 shows the 18 indicators that make of the Inclusion of Minorities Index.

**Table 1:** Indicators of the Inclusion of Minorities Index (IM)

Indicator	Source	Countries
Level of perceived discrimination among black and mulatto among self-identifying into these groups	Latinobarometer	17
% reporting that they are affected by discrimination due to skin colour or discrimination as immigrants	Latinobarometer	18
% citing “discrimination due to skin colour” and “discrimination against immigrants” affects me	Afrobarometer	18
% reporting that their economic situation is the ‘same’ as other ethnic groups in country	Afrobarometer	16
% reporting that their political situation is the ‘same’ as other ethnic groups in country	Afrobarometer	4
% reporting that their ethnic group is ‘never’ treated unfairly in country	Afrobarometer	16
Rating on uneven economic development along group lines	Fund for Peace	176
Level of ethnic tensions	Intern. Country Risk Guide	140
Level of religious tensions	Intern. Country Risk Guide	140
% who do not very much or not at all trust members of other religious groups	World Values Surveys	22
% who do not very much or not at all trust members of other nationalities	World Values Surveys	21
% citing “Being of the same social background” is very important or rather important for as successful marriage	World Values Surveys	22
Level of economic and political discrimination against minorities in country	Minorities at Risk	118
% who would reject members of another ethnic or caste group as neighbours	World Values Surveys	84
% who would reject immigrants or foreign workers as neighbours	World Values Surveys	84
% who would reject members of another religious group as neighbours	World Values Surveys	50
% who would reject other language group as neighbours	World Values Surveys	28
% who would reject Jews as neighbours	World Values Surveys	50
% who would “Prevent Labour Immigration”	World Values Surveys	50
% who are (strongly) against immigration (people of another race, from poorer countries)	European Social Survey	20
% who think “immigration is bad for economy”	European Social Survey	20
% who think “immigration is bad for cultural life”	European Social Survey	20
% who think “immigration makes country worse place to live”	European Social Survey	20
Educational disparity ethnic groups	Household surveys	75
Occupational disparity ethnic groups	Household surveys	58
Foreign/native labour participation, across all educations	OECD Factbook	26

*Source*: Institute of Social Studies, Indices of Social Development database

The dependent variable is an index for social cohesion, called Intergroup Cohesion (IC), which measures social cohesion in societies. It ranges also from 0 (no cohesion) to 1 (full cohesion). Also this index is taken from the Indices of Social Development database. Table 2 shows the 12 indicators making up the Intergroup Cohesion Index. When comparing Tables 1 and 2, one notices that there is no overlap in the indicators.

**Table 2:** Indicators of the Intergroup Cohesion Index (IC)

Indicator	Source	Countries
No of reported incidents of violent riots	Databanks	189
No of reported incidents of assassinations	Databanks	189
No of reported incidents of terrorist acts	Databanks	189
No of reported incidents of guerrilla activity	Databanks	121
Rating on likelihood of violent demonstrations	Economist Intelligence Unit	121
Rating on potential for terrorist acts	Economist Intelligence Unit	121
Rating on the ‘legacy of vengeance-seeking group grievance or group paranoia’	Fund for Peace	176
Level of civil disorder	Intern. Country Risk Guide	140
Level of internal conflict	Intern. Country Risk Guide	140
Risk of terrorism	Intern. Country Risk Guide	140
Level of ethnic minority rebellion in country	Minorities at Risk	118

*Source*: Institute of Social Studies, Indices of Social Development database

Finally, we use log of GDP per capita (lnGDPpc) as control variable, taken from World Bank’s World Development Indicators.

Table 3(a) shows the pairwise correlations between all the variables for the cross section dataset and Table 3(b) shows the correlations for the panel dataset.

**Table 3:** (a) Pairwise correlations for the cross section data, (b) pairwise correlations for the panel data

	IC	EF	LF	RF	IM	lnGDPpc
(a)						
IC	1.000000					
EF	−0.193629 (−1.554043)	1.000000				
LF	−0.247775** (−2.013781)	0.641611*** (6.586513)	1.000000			
RF	0.105086 (0.832059)	0.333814*** (2.788404)	0.423162*** (3.677462)	1.000000		
IM	0.610291*** (6.066108)	−0.413735*** (−3.578385)	−0.314960** (−2.612988)	−0.094065 (−0.743970)	1.000000	
ln GDPpc	0.281841** (2.312984)	−0.477592*** (−4.280271)	−0.524416*** (−4.849604)	−0.269286** (−2.201690)	0.240534* (1.951250)	1.000000
(b)						
IC	1.000000					
EF	−0.134121** (−2.502943)	1.000000				
LF	−0.15652*** (−2.887535)	0.674679*** (18.57594)	1.000000			
RF	0.055624 (1.028756)	0.351283*** (7.762077)	0.421457*** (9.501823)	1.000000		
IM	0.254400*** (3.710845)	−0.28491*** (−4.255680)	−0.278400 *** (−4.058030)	−0.010033 (−0.142243)	1.000000	
ln GDPpc	0.187423*** (3.518255)	−0.29069*** (−6.233599)	−0.446992*** (−10.10561)	−0.17169*** (−3.580313)	0.087053 (1.241974)	1.000000

* significance at 10 % level; ** significance at 5 % level; *** significance at 1 % level; t-statistics in brackets

The strength and significance of the pairwise correlation of our dependent variable with independent variables imply that it would be appropriate to conduct regression analysis for studying the relationship of our dependent variable with independent variables. The correlation matrices show a moderate level of correlation among our regressors both in cross sectional and panel data. Hence there seems to be less likelihood of the problem of multicollinearity.

Table 4(a) and (b) shows the summary statistics for all variables, for the cross country dataset and the panel dataset respectively.

**Table 4:** (a) Summary statistics cross-section data (2010), (b) summary statistics panel data (1990–2010)

Variable	N	Mean	SD	Min	Max
(a)					
IC	82	0.6524	0.0740	0.2040	0.7568
EF	82	0.5403	0.2418	0.0394	0.9302
LF	79	0.4810	0.3098	0.01240	0.9227
RF	80	0.4430	0.2370	0.0049	0.8603
IM	67	0.4365	0.0479	0.3107	0.5394
ln GDPpc	82	7.1799	1.08727	5.0156	9.3155
(b)					
IC	346	0.5845	0.0871	0.2040	0.7568
EF	435	0.5405	0.2383	0.0394	0.9302
LF	420	0.4695	0.3128	0.0080	0.9227
RF	435	0.4352	0.2393	0.0023	0.8603
IM	207	0.4593	0.0769	0.1726	0.8510
ln GDPpc	428	6.9332	1.0338	3.9129	9.3155

We use two estimation methods. First, we do a cross-section least squares estimation for the year 2010. Next, we make a panel for 5 years between 1990 and 2010 (data points for every 5 years). We estimate this panel dataset with random effects because the Hausman test indicated that fixed effects is not appropriate. However, it is not very likely that endogeneity is a problem. Alesina et al. (2003) have already argued that ethnic diversity is not likely to suffer from endogeneity effects, because it changes only in the long run of 20–30 years. The neighbourhood-level study by Stolle et al. (2008) found no endogeneity effects of diversity, confirming the assumption that ethnic diversity can be treated as an exogenous variable. A similar argument can be made for Inclusion of Minorities, which is likely to change only slowly over time—the reason why the data is collected not on an annual basis but with five-year intervals.

In the Appendix, we report the results of fixed effects estimations for the panel data, just for comparison with our random effects results (Table 7). The fixed effects results are even stronger than the random effects results. For all three variations of fractionalization (ethnic, linguistic and religious) the sign of the parameter is positive and the parameter value is statistically significant, whereas the sign for inclusion of minorities is also positive and statistically significant in all three estimations. This suggests that when controlled for endogeneity through country fixed effects, the impact of fractionalization on growth is positive rather than negative, when we control for social exclusion. At the same time, the results indicate that the effect of inclusion of minorities on growth is positive and strong.

Although the empirical literature often uses panel data with ethnic fractionalization as the explanatory variable, we emphasize the results of the cross-country analysis, because the data for fractionalization is available for 1 year only (not only in our study but also in the literature that we refer to). Hence, panel estimation would use variation over time whereas the key variable of interest does not have such variation. Finally, we will also do robustness checks for the ethnic diversity variable using linguistic and religious diversity in the cross-section estimation.

## Results

Table 5(a) shows the results of the cross-section estimation with Intergroup Cohesion (IC) as the dependent variable. Model 1 only includes Ethnic Fractionalization (EF) and income (lnGDPpc) as a control variable. Model 2 adds Inclusion of Minorities (IM). Models 3 and 4 show the results of the robustness tests for Linguistic Fractionalization (LF) and Religious Fractionalization (RF) respectively.

**Table 5:** (a) Results of cross-section estimation: dependent variable Intergroup Cohesion, (b) results of the random effects panel estimation; dependent variable Intergroup Cohesion

Variable	Model 1	Model 2	Model 3	Model 4
(a)				
EF	−0.04038 (−0.9796) [1.1116]	0.06671 (1.4732) [1.4528]		
LF			0.00297 (0.0833) [1.4193]	
RF				0.08619** (2.0940) [1.0711]
IM		1.19813*** (5.9464) [1.2326]	1.13274*** (5.4328) [1.0952]	1.12763*** (5.9173) [1.0461]
ln GDPpc	0.02107** (2.2416) [1.1116]	0.01882** (2.0103) [1.2576]	0.01391 (1.3135) [1.3595]	0.01846** (2.0616) [1.1142]
Constant	0.52139*** (6.6750)	−0.04795 (−0.3874)	0.05233 (0.4209)	−0.01712 (−0.1703)
R^2^	0.09402	0.41862	0.39028	0.43654
F-statistic	4.0996***	15.1209***	12.5886***	15.7529***
N	82	67	63	65
(b)				
EF	−0.01361 (−0.5029) [1.1199]	−0.00114 (−0.0340) [1.2209]		
LF			0.01004 (0.3724) [1.4338]	
RF				0.10453*** (3.4690) [1.0869]
IM		0.23295*** (2.6635) [1.0587]	0.24130*** (2.7349) [1.0596]	0.25495*** (3.1189) [1.0060]
ln GDPpc	0.01845*** (3.0436) [1.1199]	0.01332* (1.8337) [1.1587]	0.01549* (1.8917) [1.3649]	0.01940*** (2.9320) [1.0909]
Constant	0.46425*** (9.3278)	0.38445*** (5.0460)	0.36062*** (4.4310)	0.28405*** (4.4724)
R^2^	0.03268	0.05339	0.05573	0.11245
F-statistic	5.6924***	3.6472**	3.6589**	8.0663***
N	340	198	190	195

* significance at 10 % level; ** significance at 5 % level; *** significance at 1 % level; t-statistics in round brackets and VIF in square brackets

Finally, Table 5(b) shows the results of the random effects panel estimation, in which the same data for ethnic fractionalization are used in all 5 years.

We have reported t-statistics to describe the significance of coefficients of our independent variables. F-statistics have been reported for each model. We have also added VIF statistics to check for the possible existence of multicollinearity in our models. The significance of F-statistics in all of our models confirms the joint significance of independent variables in our models. It implies that models used for our empirical analysis are appropriate. Reported VIF statistics are closer to 1 and hence rule out the possibility of multicollinearity. Finally, as indicated above, the Appendix shows a table with results for a fixed effects estimation of the panel data, controlling for possible endogeneity (Table 7). Moreover, the Appendix also includes stepwise regressions, indicating that inclusion of minorities is a relevant explanatory variable both in the cross section and in the panel regressions (Tables 8, 9).

The models in the cross country estimations show that as soon as Inclusion of Minorities is added, the model fit increases substantially as compared to model 1. A similar effect can be found for the panel estimations, but the size of R square is much lower for all the models as compared to the cross country models. Inclusion of Minorities is statistically significant in all the three models in which it is included, both in the cross country estimations and the panel estimations. To the contrary, ethnic fractionalization and linguistic fractionalization do not show statistically significant effects. Religious fractionalization shows in both types of estimations statistically significant parameters, but in both cases with an unexpected positive sign. We discuss the findings in more detail in the next section.

## Discussion

The results of the cross-section and panel estimations are quite similar. All models show, as expected, positive and statistically significant results for GDP per capita. More developed economies tend to have higher levels of Intergroup Cohesion, most likely due to better governance systems and higher levels of redistribution between social groups.

Table 5(a), with the cross-section results, points out that model 1 shows a small negative effect of Ethnic Fractionalization (EF) on Intergroup Cohesion (IC): a 0.10 increase in Ethnic Fractionalization is associated with a 0.004 decline in Intergroup Cohesion (both on a scale of 0–1). The parameter, however, is not statistically significant. Model 2 adds the social exclusion measure and shows that now the sign for Ethnic Fractionalization is positive, but not statistically significant. Inclusion of Minorities (IM), however, has a statistically significant positive effect: a 0.10 increase in Inclusion of Minorities (10 % of the scale) is associated with a 0.12 increase in Intergroup Cohesion, which is a substantive effect, larger than one standard deviation in Intergroup Cohesion. Models 3 and 4 show the results of the robustness tests with two alternative variables for Ethnic Fractionalization. Model 3 shows that Linguistic Fractionalization (LF) has a positive but no statistically significant effect on Intergroup Cohesion, whereas Inclusion of Minorities has a relatively large positive and statistically significant effect, as in model 2. Model 4 shows a slightly different result. Both Religious Fractionalization (RF) and the Inclusion of Minorities have positive and statistically significant parameters. But the size effect of inclusion is much larger than that of religious diversity: 0.10 increase in Religious Fractionalization is associated wit 0.009 increase in Intergroup Cohesion (1 % of the standard deviation of Intergroup Cohesion). Whereas 0.10 increase in Inclusion of Minorities is associated with 0.11 increase in Intergroup Cohesion, which is more than a standard deviation.

Table 5(b) shows the results of the panel estimation for the period 1990–2010. Both in model 1 and model 2, the signs of the parameters for Ethnic Fractionalization are negative, as is found in much of the empirical literature with panel estimations. Remember that the fractionalization data are available for 1 year only, so that there is no variation over time for this variable. The parameters for Ethnic Fractionalization are not statistically significant. Model 2 shows that the effect of Inclusion of Minorities is substantive. 0.10 increase in Inclusion of Minorities is associated with sizeable 0.23 increase in Intergroup Cohesion. Models 3 and 4 show again the results for the robustness tests. In model 3 we see that Linguistic Fractionalization (LF) is not statistically significant. In model 4, we find that Religious Fractionalization (RF) shows a positive and statistically significant correlation with Intergroup Cohesion, very similar as in the results of the cross-section analysis. Models 2, 3 and 4 show very similar parameters for Inclusion of Minorities, all quite large and statistically significant.

Taken together, the results of the cross-section analysis and the panel analysis indicate that Ethnic Fractionalization and Linguistic Fractionalization have no statistically significant effect on Intergroup Cohesion, while Religious Fractionalization has a very small positive effect. Both types of estimations also point out that Inclusion of Minorities has a strong positive effect on Intergroup Cohesion. When we compare this effect with the income effect, we find that 10 % higher GDP per capita has a much smaller impact on Intergroup Cohesion than a 0.10 point increase (for example from 0.30 to 0.40) along the scale of Inclusion of Minorities. In other words, our results suggest that for social cohesion it is not income or diversity, which matters, but the way in which social groups relate to each other.

## Conclusion

We find that horizontal inequality has a statistically significant and substantial negative impact on social cohesion. Vertical inequality concerns unequal earnings, whether based on differences in human capital, demand for skills, discrimination, or exploitation. In most developing countries, the vast majority of the population earns a relatively low income, irrespective to which ethnic group they belong. This is the case for Hutus and Tutsis in Rwanda, for Hindus and Muslims in India, and for Oromo and Tigray in Ethiopia, for example. Each of these ethnic, linguistic or religious groups has a large underclass of very poor households, so that vertical inequality is not the major issue distinguishing these groups. Horizontal inequality is different, because it occurs along the lines of the social groups themselves—not in terms of income but in terms of identities, rights, opportunities, capabilities, and voice. In the case of horizontal inequalities, complete groups of people are largely excluded from society and the economy. This affects their access to assets, market opportunities and public goods. Such social exclusion creates not merely *differences* in economic benefits, as is the case for vertical inequality, but *exclusion* from important parts of the economy, which is a more fundamental constraint affecting social cohesion and in turn economic growth.

Our results indicate that once controlled for horizontal inequality, ethnic diversity has no statistically significant negative impact on social cohesion. At the same time, social exclusion, which we measured with an index of inclusion of minorities, shows a positive relatively large and statistically significant effect on social cohesion, indicating that social exclusion reduces social cohesion. We conclude that future cross country studies of social cohesion are likely to benefit from including variables for social exclusion, as a more accurate measure of how diversity contributes to the exclusion of social groups in a society. We also suggest that growth regressions might include variables of social cohesion and social exclusion, in order to test the complete social economic model of diversity and growth, as presented in this article. For now, we have indicated that the mechanism linking diversity and growth, namely social cohesion, is influenced more by social exclusion than by ethnic diversity.

## Appendix

See Tables 6, 7, 8 and 9.

**Table 6:** Country lists

Countries in cross section data	Models 1, 2, 3, or 4	Countries in panel data	Models 1, 2, 3, or 4
Albania	1, 2, 3, 4	Albania	1, 2, 3, 4
Angola	1, 2, 3, 4	Angola	1, 2, 3, 4
Argentina	1, 2, 3, 4	Argentina	1, 2, 3, 4
Armenia	1, 2, 3, 4	Armenia	1, 2, 3, 4
Azerbaijan	1, 2, 3, 4	Azerbaijan	1, 2, 3, 4
Bangladesh	1, 2, 3, 4	Bangladesh	1, 2, 3, 4
Belarus	1, 2, 3, 4	Belarus	1, 2, 3, 4
Bolivia	1, 2, 3, 4	Bolivia	1, 2, 3, 4
Bosnia and Herzegovina	1, 2, 3, 4	Bosnia and Herzegovina	1, 2, 3, 4
Brazil	1, 2, 3, 4	Brazil	1, 2, 3, 4
Bulgaria	1, 2, 3, 4	Burundi	1
Burkina Faso	1, 2	Bulgaria	1, 2, 3, 4
Cambodia	1	Burkina Faso	1, 2, 3, 4
Cameroon	1, 2, 3, 4	Cambodia	1
Chad	1	Cameroon	1, 2, 3, 4
China	1, 2, 3, 4	Chad	1
Colombia	1, 2, 3, 4	China	1, 2, 3, 4
Congo, Dem. Rep.	1, 2, 3, 4	Colombia	1, 2, 3, 4
Congo, Rep.	1, 2, 3, 4	Congo, Dem. Rep.	1, 2, 3, 4
Costa Rica	1, 2, 3, 4	Congo, Rep.	1, 2, 3, 4
Côte d’Ivoire	1	Costa Rica	1, 2, 3, 4
Dominican Rep.	1, 2, 3, 4	Côte d’Ivoire	1
Ecuador	1, 2, 3, 4	Djibouti	1, 2, 3, 4
Egypt	1, 2, 3, 4	Dominican Rep.	1, 2, 3, 4
El Salvador	1, 2	Ecuador	1, 2, 3, 4
Ethiopia	1, 2, 3, 4	Egypt	1, 2, 3, 4
Gabon	1	El Salvador	1, 2, 4
Gambia	1	Ethiopia	1, 2, 3, 4
Georgia	1, 2, 3, 4	Gabon	1
Ghana	1, 2, 3, 4	Gambia	1
Guinea	1, 2, 3, 4	Georgia	1, 2, 3, 4
Guinea-Bissau	1	Ghana	1, 2, 3, 4
Guyana	1, 2, 3, 4	Guinea	1, 2, 3, 4
Haiti	1	Guinea-Bissau	1
Honduras	1, 2, 3, 4	Guyana	1, 2, 3, 4
Hungary	1, 2, 3, 4	Haiti	1
India	1, 2, 3, 4	Honduras	1, 2, 3, 4
Indonesia	1, 2, 3, 4	Hungary	1, 2, 3, 4
Jordan	1, 2, 3, 4	India	1, 2, 3, 4
Kazakhstan	1, 2, 3, 4	Indonesia	1, 2, 3, 4
Kenya	1, 2, 3, 4	Jordan	1, 2, 3, 4
Kyrgyzstan	1, 2, 3, 4	Kazakhstan	1, 2, 3, 4
Lao	1	Kenya	1, 2, 3, 4
Liberia	1, 2, 3, 4	Kyrgyzstan	1, 2, 3, 4
Madagascar	1, 2, 3, 4	Lao	1
Malawi	1, 2, 3, 4	Liberia	1, 2, 3, 4
Mali	1, 2, 3, 4	Madagascar	1, 2, 3, 4
Mauritania	1	Malawi	1, 2, 3, 4
Mexico	1, 2, 3, 4	Mali	1, 2, 3, 4
Moldova	1, 2, 3, 4	Mauritania	1
Mongolia	1	Mexico	1, 2, 3, 4
Mozambique	1, 2, 3, 4	Moldova	1, 2, 3, 4
Namibia	1, 2, 3, 4	Mongolia	1
Nicaragua	1, 2, 3, 4	Mozambique	1, 2, 3, 4
Niger	1, 2, 3, 4	Namibia	1, 2, 3, 4
Nigeria	1, 2, 3, 4	Nicaragua	1, 2, 3, 4
Pakistan	1, 2, 3, 4	Niger	1, 2, 3, 4
Panama	1, 2, 3, 4	Nigeria	1, 2, 3, 4
Paraguay	1, 2, 3, 4	Pakistan	1, 2, 3, 4
Peru	1, 2, 3, 4	Panama	1, 2, 3, 4
Philippines	1, 2, 3, 4	Paraguay	1, 2, 3, 4
Romania	1, 2, 3, 4	Peru	1, 2, 3, 4
Rwanda	1, 2	Philippines	1, 2, 3, 4
Senegal	1, 2, 3, 4	Romania	1, 2, 3, 4
Serbia	1, 2	Rwanda	1, 2, 4
Sierra Leone	1, 2, 3, 4	Senegal	1, 2, 3, 4
South Africa	1, 2, 3, 4	Serbia	1, 2
Sri Lanka	1, 2, 3, 4	Sierra Leone	1, 2, 3, 4
Suriname	1, 2	South Africa	1, 2, 3, 4
Tajikistan	1, 2	Sri Lanka	1, 2, 3, 4
Tanzania	1, 2, 3, 4	Suriname	1
Thailand	1, 2, 3, 4	Syria	1, 2, 3, 4
Tunisia	1, 2	Tajikistan	1
Turkey	1, 2, 3, 4	Tanzania	1, 2, 3, 4
Turkmenistan	1, 2	Thailand	1, 2, 3, 4
Uganda	1, 2, 3, 4	Togo	1
Ukraine	1, 2, 3, 4	Tunisia	1
Uzbekistan	1	Turkey	1, 2, 3, 4
Venezuela	1, 2, 3, 4	Turkmenistan	1
Viet Nam	1, 2, 4	Uganda	1, 2, 3, 4
Yemen	3, 4	Ukraine	1, 2, 3, 4
Zambia	1, 2, 3, 4	Uzbekistan	1
Zimbabwe	1, 2, 3, 4	Vanuatu	1, 2, 3, 4
		Venezuela	1, 2, 3, 4
		Viet Nam	1, 2, 3, 4
		Zambia	1, 2, 3, 4
		Zimbabwe	1, 2, 3, 4

**Table 7:** Fixed effects panel estimations: dependent variable Intergroup Cohesion

Variable	Model 1	Model 2	Model 3
EF	1.496169** (2.61)		
LF		0.5587069** (2.39)	
RF			0.8731642** (2.43)
IM	0.3080344** (2.57)	0.3010979** (2.52)	0.2988754** (2.52)
ln GDPpc	0.1593863*** (4.99)	0.1479289*** (4.62)	0.1497964*** (4.71)
Constant	−1.12732*** (−2.90)	−0.7254947** (−2.48)	−1.128974*** (−2.65)
R^2^	0.5508	0.5571	0.5547
F-statistic	2.22***	2.29***	2.26***
N	198	190	195

* significance at 10 % level; ** significance at 5 % level; *** significance at 1 % level; t-statistics in brackets

**Table 8:** Stepwise regression cross country dataset

Dependent variable: Intergroup Cohesion
Method: stepwise regression
Included observations: 65
Number of search regressors: 5
Selection method: stepwise forwards
Stopping criterion: *p* value forwards/backwards = 0.05/0.05

Variable	Coefficient	SE	t-statistic	Prob.*
RF	0.087773	0.037608	2.333881	0.0229
ln GDPpc	0.017840	0.007565	2.358186	0.0215
IM	1.096429	0.134352	8.160875	0.0000
R^2^	0.435938	Mean dependent var		0.648658
Adjusted R^2^	0.417743	SD dependent var		0.093789
SE of regression	0.071567	Akaike info criterion		−2.391316
Sum squared resid	0.317552	Schwarz criterion		−2.290960
Log likelihood	80.71777	Hannan-Quinn criter.		−2.351719
Durbin–Watson stat	1.856391

**Table 9:** Stepwise regression panel dataset

Dependent variable: Intergroup Cohesion
Method: stepwise regression
Included observations: 195
Number of search regressors: 5
Selection method: stepwise forwards
Stopping criterion: *p* value forwards/backwards = 0.05/0.05

Variable	Coefficient	SE	t-statistic	Prob.*
RF	0.151623	0.026226	5.781428	0.0000
ln GDPpc	0.039183	0.004310	9.091299	0.0000
IM	0.505551	0.071569	7.063779	0.0000
R^2^	0.045430	Mean dependent var		0.584207
Adjusted R^2^	0.035486	SD dependent var		0.090777
SE of regression	0.089152	Akaike info criterion		−1.981680
Sum squared resid	1.526036	Schwarz criterion		−1.931326
Log likelihood	196.2138	Hannan-Quinn criter.		−1.961292
Durbin–Watson stat	1.197341			

## Footnotes

1
www.IndSocDev.org.
